# Shear Stress Quantification in Tissue Engineering Bioreactor Heart Valves: A Computational Approach

**DOI:** 10.3390/jfb15030076

**Published:** 2024-03-20

**Authors:** Raj Dave, Giulia Luraghi, Leslie Sierad, Francesco Migliavacca, Ethan Kung

**Affiliations:** 1 Department of Mechanical Engineering, Clemson University, Clemson, SC 29634, USA; rajdave95@gmail.com; 2 Department of Chemistry, Material and Chemical Engineering, Politecnico di Milano, 20133 Milan, Italy; 3 Aptus, LLC, Clemson, SC 29631, USA; 4 Department of Bioengineering, Clemson University, Clemson, SC 29634, USA

**Keywords:** wall shear stress quantification, TEHV, FSI, CFD, computational model

## Abstract

Tissue-engineered heart valves can grow, repair, and remodel after implantation, presenting a more favorable long-term solution compared to mechanical and porcine valves. Achieving functional engineered valve tissue requires the maturation of human cells seeded onto valve scaffolds under favorable growth conditions in bioreactors. The mechanical stress and strain on developing valve tissue caused by different pressure and flow conditions in bioreactors are currently unknown. The aim of this study is to quantify the wall shear stress (WSS) magnitude in heart valve prostheses under different valve geometries and bioreactor flow rates. To achieve this, this study used fluid–structure interaction simulations to obtain the valve’s opening geometries during the systolic phase. These geometries were then used in computational fluid dynamics simulations with refined near-wall mesh elements and ranges of prescribed inlet flow rates. The data obtained included histograms and regression curves that characterized the distribution, peak, and median WSS for various flow rates and valve opening configurations. This study also found that the upper region of the valve near the commissures experienced higher WSS magnitudes than the rest of the valve.

## 1. Introduction

Heart valve disease affects 2.5% of the US adult population, with a higher incidence in older individuals. Currently, treatment options are limited and surgery is recommended for severe, symptomatic cases [1,2]. Valve replacement options include mechanical and bioprosthetic heart valves, but both have significant drawbacks. Mechanical heart valves are durable but require lifelong anti-coagulation therapies and result in non-physiological flow patterns. They have a higher risk of major adverse cardiovascular complications, particularly major bleeding [3]. Bioprosthetic heart valves can overcome some of the limitations of mechanical heart valves, but their durability is limited to 10–15 years, making them unsuitable for younger patients [4,5]. In pediatric patients, both types of valve prostheses require frequent resizing surgeries due to their inability to remodel and adapt to growth [6].

Tissue engineering presents a promising opportunity to create heart valve prostheses that are superior to the existing options. A commonly used approach for developing tissue-engineered heart valves involves pre-seeding cells on a scaffold and maturing them in a bioreactor under controlled physiological conditions [7,8]. A properly matured tissue-engineered heart valve (TEHV) seeded with human cells has the potential to grow, repair, and remodel once implanted. To achieve functional valve tissue, it is important to subject the seeded human cells to physiological conditions for maturation. There are different types of bioreactors that can produce in vitro environments with different flow rates, pressures, temperatures, and oxygen diffusion to condition TEHVs [9]. Bioreactors can be broadly classified into flow-based, strain-based whole valve conditioning, and isolated cusp stimulation bioreactors [9,10]. The optimal maturation protocol for nurturing seeded cells on a valve scaffold is still unknown, and the mechanical cues resulting from the flow rate and pressure differential are difficult to quantify. TEHVs are usually conditioned with between 2- and 6-week cycles with mixed outcomes [8,9,11,12].

Previous attempts on valve conditioning have highlighted the importance of understanding the magnitude of wall shear stress (WSS) as the flows within the bioreactor during the conditioning protocol are progressively adjusted. VeDepo et al. investigated the potential for leaflet matrix restoration and repopulation using bone marrow-derived mononuclear cells; human aortic valves were decellularized and seeded with marrow-derived mononuclear cells and the test samples were subjected to different conditioning protocols. The study found that extended conditioning protocols (i.e., mechanical cues) led to the retraction of valve leaflets, suggesting that an extended period of mechanical conditioning is not feasible [13]. The findings of VeDepo et al. suggest that the application of excessive WSS could potentially have a negative impact on the development and function of TEHVs. Kennamer et al. sought to investigate whether stem cells would differentiate into valvular interstitial cells when subjected to mechanical stimuli. The test group samples were subjected to mechanical cues for four weeks of in vitro bioreactor conditioning, and the study found that most of the seeded cells had died, resulting in a large island of cell debris [14]. The study concluded by emphasizing the sensitivity of stem cells to mechanical cues and the need for careful, progressive adaptation to these cues. 

The regulation of homeostasis in the endothelial cell lining depends significantly on WSS [15,16]. Several ex vivo studies [17,18,19] have shown that changes in WSS can lead to the valve becoming diseased. Although some studies [20,21,22,23,24] have provided an estimate of the WSS in the aortic valve during normal flow conditions, there is a lack of information about the WSS distribution in TEHVs and how it changes as flow increases during conditioning [25]. Previous studies on heart valve WSS also assumed aortic valve geometries with sinuses (which may not be present in bioreactors) and considered blood-like properties to model fluid properties; however, culture media fluids in bioreactors have properties more similar to water. There is a need obtain WSS estimates in TEHVs at various bioreactor flow rates. This knowledge would benefit researchers working on conditioning protocols, enabling them to optimize flow ramp up rates and potentially gain a better understanding of WSS-related cell adhesion and growth.

The objective of this study is to quantify WSS in TEHVs during the maturation process in a bioreactor. To achieve this, we used fluid–structure interaction (FSI) simulations to generate valve geometries at different points in the pulsatile flow cycle; we then conducted computational fluid dynamics (CFD) simulations with these valve geometries to quantify shear stresses on the valve leaflets at multiple flow rates. This study focuses on the aortic valve with annulus diameters of 12.3 mm, 18.45 mm, and 24.6 mm, representing pediatric, adolescent, and adult heart valves, respectively; our findings suggest that the data we report may be generalized to valves of other different sizes.

## 2. Materials and Methods

The dynamics of heart valves are influenced by the fluid flow and pressure differences across them. FSI simulations can model valve dynamics by coupling the loading on the valve caused by the fluid to the disturbance on the fluid by the valve motion. To achieve this, FSI simulations solve the continuum equations for the solid valve, as well as the continuity and Navier–Stokes equations for the fluid, to model the two-way fluid–structure interaction (between the valve and the fluid). Two approaches exist in modeling the FSI: (1) the monolithic approach, which uses a single algorithm to solve the fluid and solid domain equations; (2) the partitioned approach, which solves the two sets of domain equations in separate algorithms and uses a coupling algorithm to balance the forces exchanged and continuity of the domain between the fluid and structural regions. This study uses the partitioned method [26,27], which has two distinct, overlapping meshes. We adopted the non-boundary fitted method, where the fluid mesh remains fixed while the solid mesh deforms and intersects the fluid mesh, resulting in significantly reduced computational expense compared to traditional Arbitrary Lagrangian Eulerian methods where both meshes must deform. However, the limitation of this approach is that the fluid mesh being homogenous prohibits near-wall mesh refinement for obtaining accurate WSS quantification. We overcame this limitation of the FSI by extracting open valve geometries at different time points during systole, then using these geometries to perform subsequent CFD simulations which allow for boundary layer meshing to obtain detailed WSS. The details of both the FSI and the CFD phase are outlined in the sections to follow. 

### 2.1. FSI Simulations

**Figure 1:**
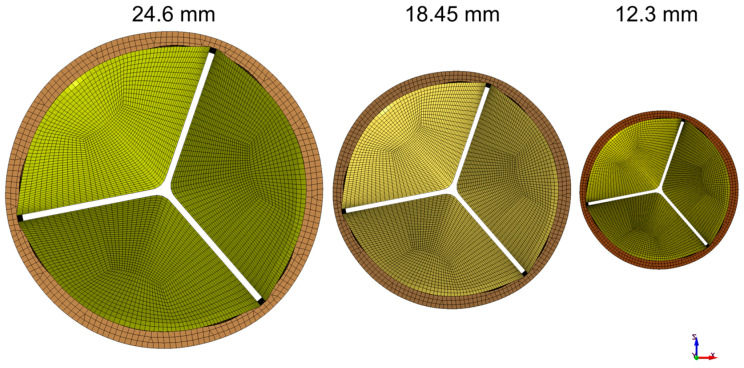
Valves representing pediatric, adolescent, and adult sizes: 12.3, 18.45, and 24.6 mm, respectively.

We use an idealized aortic valve with a uniform leaflet thickness of 0.4 mm, identical deformable leaflets, and a rigid corona with a ring at the base. For the reference model the valve diameter and the ring external diameter were 24.6 mm and 28 mm, respectively; the total height of the geometry was 17 mm. The geometries for the smaller-sized valve (12.30 and 18.45 mm) were obtained by scaling the reference model. Three idealized valves represented pediatric, adolescent, and adult sizes 12.3, 18.45 and 24.6 mm, respectively (Figure 1). The non-boundary fitted FSI simulation we used is based on the study by Luraghi et al. [27] and was validated experimentally for a polymeric heart valve (similar to a styrenic block copolymer) in [28]. The study [28] demonstrated the accuracy of FSI simulation in estimating the valve kinematics, such as the geometric orifice area (GOA), against experimental results. The material model for the leaflet is linear elastic for the working strain range, with an elastic modulus of 3 MPa, a Poisson’s ratio of 0.40, and a density of 1100 kg/m^3^. The Young’s modulus utilized represents the material property of the TEHV prior to undergoing dynamic conditioning [29]. The fluid in the FSI simulation is modeled as a Newtonian fluid with a density of 1060 kg/m^3^ and a dynamic viscosity of 3 cP, similar to that in Luraghi et al. [27]. A physiological pressure difference is the driving boundary condition. We conducted the simulations over two cardiac cycles, each with a duration of 0.8 s, and extracted the results from the second cycle. All FSI simulations were performed on 8 cores of a 14-core Intel-MPI Xeon64 processor using the commercial finite element solver LS-Dyna Release 11 (ANSYS Inc., Canonsburg, PA, USA). 

### 2.2. GOA and Flow Rate Selection

**Figure 2:**
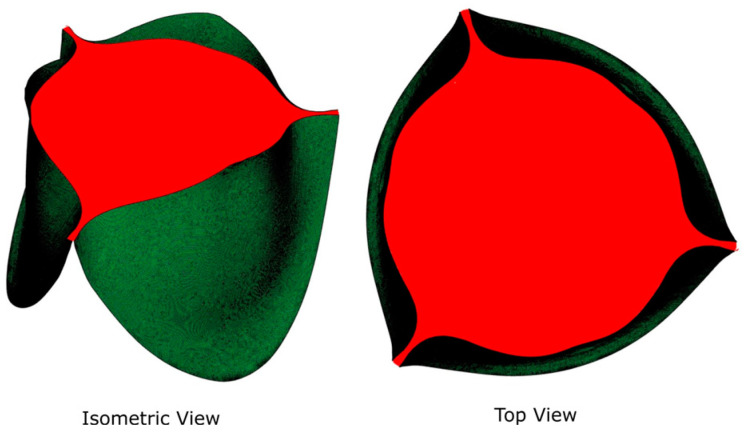
Isometric and planform (top) views of GOA.

**Figure 3:**
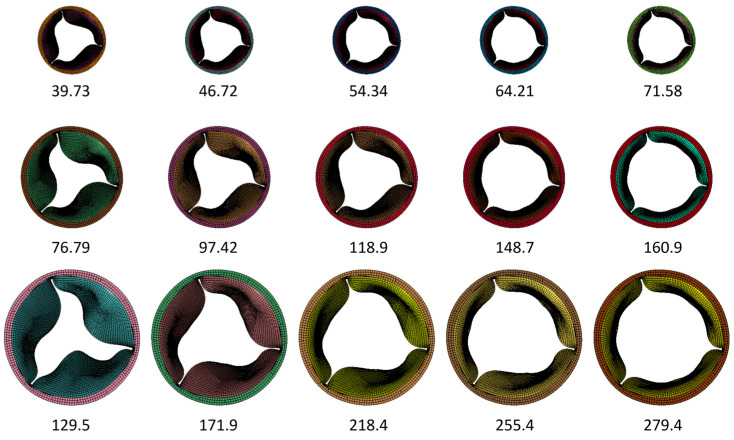
Summary of the geometries included in the CFD simulation to quantify WSS. Numbers represent the GOA in mm^2^.

We selected five GOAs, ranging from 45% to 100% of GOA (normalized to the maximal values during the cycle), from each of the three FSI simulations for subsequent CFD simulation (Table 1). The GOA is the anatomical area of the aortic valve orifice and is obtained from image planimetry [30]. Planform views (Figure 2) of the valve with dimensional references were imported into Fiji ImageJ [31] and measured for GOA. The GOA for the 15 geometries (5 GOA × 3 Valve sizes) selected for this study approximately range from 39 to 279 mm^2^ (Figure 3).

For each geometry extracted from the FSI simulation, we performed multiple CFD simulations with a range of flow rates. The purpose of this was to collect data corresponding to different potential combinations of GOAs and flow rates, considering the progressive increases in flow rates in a typical TEHV conditioning protocol in the bioreactor. Prior studies [20,21,32,33] reported instantaneous peak flow rates ranging from 20 to 25 L/min for an average cardiac output of 5 L/min. We thus selected the following flow rates for the CFD simulations: 5, 11, 18, 24, and 30 L/min, which encompass the wide range of flow rates that could occur in the bioreactor. Table 2 summarizes the flow and GOA combinations we included in this study.

### 2.3. CFD Simulations

The extracted valve geometries were processed in a pre-processor package, Hypermesh (Altair Engineering, Inc., Troy, MI, USA), to create the two-dimensional surface mesh for use in CFD. This surface mesh consists of the valve housed inside a cylinder of diameter 32.4 mm, analogous to how a TEHV is housed inside a bioreactor. Using the tip of the valve as a reference point, the inlet to the valve was situated 60 mm upstream, representing the typical short bioreactor inlet, and the outlet was located 1200 mm downstream to ensure numerical stability. The geometric model thus formed an enclosed fluidic volume with the valve situated within. The size of the domain was the same for all valves we simulated.

This surface mesh was imported into ANSYS FLUENT 19 R2 (ANSYS Inc., Canonsburg, PA, USA) to discretize the flow volume with three-dimensional elements and generate 12 boundary layers on the ventricular side of the valve using ANSYS FLUENT MESHING. The volumetric mesh is composed of hexahedral elements at the center with transition into polyhedral elements near the surface (poly-hexcore). A grid convergence study was carried out for the largest GOA of each valve (see Supplemental Materials).

In the CFD simulations, we modeled the fluid properties to resemble water at 37 °C with a density of 993.3 kg/m^3^ and a dynamic viscosity of 6.914 × 10^−4^ Pa·s. Prior studies, [20,21,24,32], in an attempt to quantify stresses under physiological conditions in vivo, modeled the fluid to resemble blood (density 1040~1060 kg/m^3^, dynamic viscosity 3.5 cP). However, our viscometry measurement (using Cannon 9721-R59 viscometer, CANNON Instrument Company, State College, PA, USA) of the culture media indicated that the media closely resembled water (density 1008.5 kg/m^3^, dynamic viscosity 9.141 × 10^−4^ Pa·s). Thus, we decided to model the fluid to resemble water at 37 °C. A constant magnitude velocity (plug type) was prescribed at the inlet. This is justified as most bioreactors have short inlet lengths. The plug type inlet velocities were derived from the volumetric flow rate based on the continuity equation Q = V ∗ A, where Q is the volumetric flow rate, V is the mean velocity, and A is the cross-sectional area of the cylinder domain (824.48 mm^2^). At the outlet, an “outflow” boundary condition was prescribed, which is recommended when the downstream pressure and velocity are unknown [33]. The solution method uses the transient pressure-based solver with a laminar model with cell-based least squares for the gradient spatial discretization. The pressure and momentum equations use second order and second order upwind discretization, respectively. The transient formulation is a bounded second order implicit scheme. The solution method is a Pressure-Implicit with Splitting of Operators (PISO) form of pressure–velocity coupling. Relaxation factors for the solvers were set to default, while the target residuals of continuity and velocity were set to 1 × 10^−6^. The required time step size was based upon the equation Δt = L/(3V), where L and V are the characteristic length and velocity, respectively. The time step size was set to 0.01 s to standardize all the simulations. All simulations use the standard form of solution initialization based on the inlet velocity and have a simulated flow time of at least 20 s. The simulations were performed on a mixture of 24 and 28 core computing nodes on the Palmetto cluster, which is Clemson University’s primary high performance computing resource. The WSS histograms were obtained by exporting cell centered WSS data from all the leaflets. The WSS was measured on the entire leaflet surface.

## 3. Results

### 3.1. The 24.6 mm Valve

**Figure 4:**
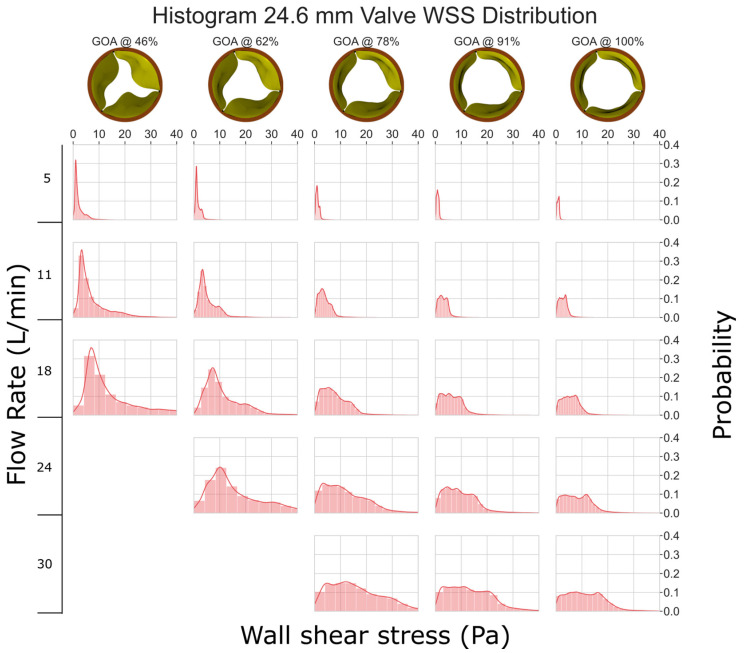
WSS histogram for the 24.6 mm valve. Flow rate is arranged in the vertical direction and increases down the columns. % GOA is arranged in the horizontal direction. The area of each histogram is individually normalized to 1.

When the flow rate is low (between 5 and 11 L/min), the WSS histogram has a shape like an impulse function (Figure 4). This implies that the entire leaflet surface is subjected to similar levels of WSS. As the flow rate and GOA increase, the histogram tends to become more diffused in shape; this implies that different areas of the valve experience drastically different levels of WSS. Despite this, all the histograms are right-skewed, indicating that most of the leaflets experience low levels of WSS (less than 40 Pa). Previous research, such as [34,35], has demonstrated that shear stresses greater than 40 Pa can harm the endothelial lining.

### 3.2. The 18.45 mm Valve

**Figure 5:**
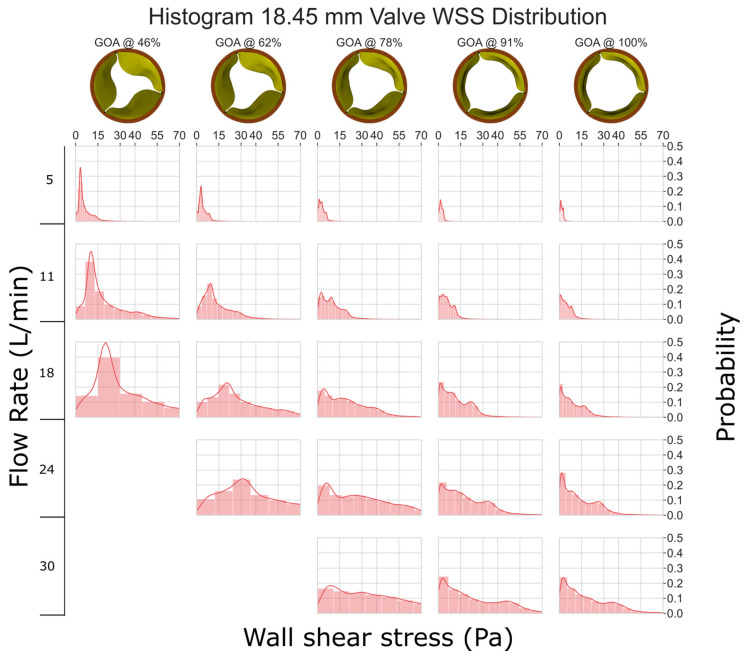
WSS histogram for the 18.45 mm valve. Flow rate is arranged in the vertical direction and increases down the columns. % GOA is arranged in the horizontal direction. The area of each histogram is individually normalized to 1.

As compared to Figure 4, the smaller valve size (GOA) and higher velocities lead to increased stresses in the 18.45 mm valve (Figure 5). The graph clearly shows that as GOA decreases and the flow rate remains constant, WSS increases. Although most of the leaflets experience less than 40 Pa WSS, some instances at a low GOA and a high flow rate show WSS between 40 and 70 Pa, which can potentially damage cells ([34,35]).

### 3.3. The 12.3 mm Valve

**Figure 6:**
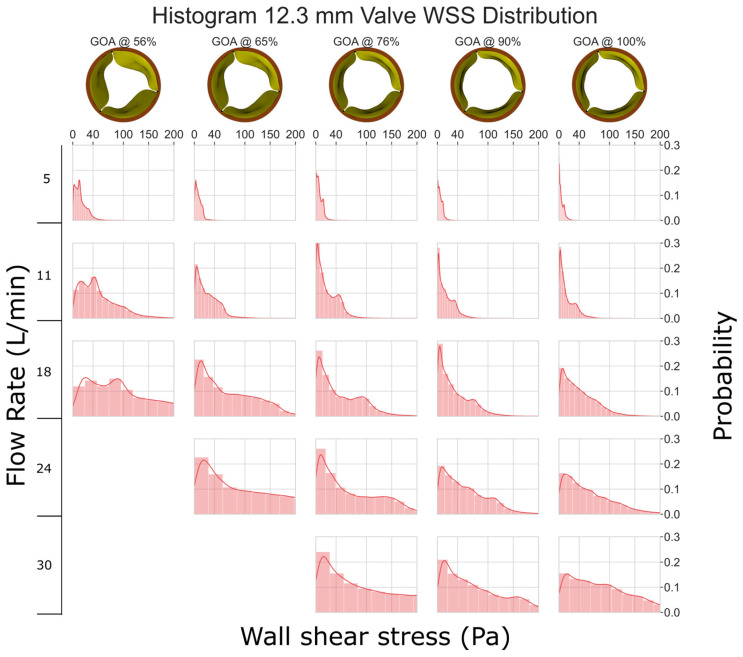
WSS histogram for the 12.3 mm valve. Flow rate is arranged in the vertical direction and increases down the columns. % GOA is arranged in the horizontal direction. The area of each histogram is individually normalized to 1.

In Figure 6, it is evident that the 12.3 mm valve experiences considerably higher levels of WSS when compared to the other valves. At a flow rate of 5 L/min, the WSS is mostly <40 Pa. However, at higher flow rates, we consistently observed the WSS exceeding 100 Pa, which has the potential to cause erosion of the endothelial cells [34,35].

### 3.4. WSS Distribution within the Leaflet

**Figure 7:**
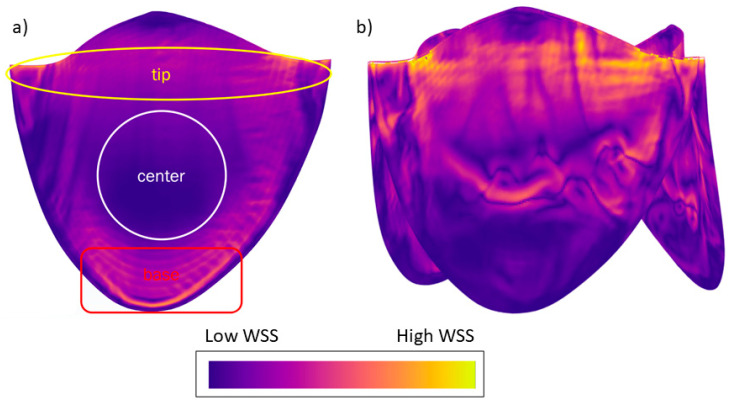
(**a**) Typically observed WSS pattern in the valve simulations. (**b**) Altered WSS pattern due to separation of flow from the valve surface for smaller valves with high flow rates. WSS legend: orange indicates regions of higher WSS and deep blue regions of lower WSS. The image is presented for illustrative purposes to depict the typical distribution of WSS observed during this study; therefore, specific values are not shown.

We typically saw three regions of WSS in the simulation results (Figure 7a). The tip of the leaflet experienced high-magnitude WSS; especially near the corners of the leaflets, the highest magnitudes were observed due to fluid acceleration resulting from converging geometry. At the center of the leaflet, we observed a low-magnitude region of WSS. At the base of the leaflet, moderate-to-elevated WSSs were observed due to fluid re-circulation. This pattern is consistent across almost all flow rates and geometries, except for high flow rates of the 12.3 and 18.45 mm valves, where we observed increased stresses at the center due to flow separation (Figure 7b).

### 3.5. Regression Models of Shear Stress

We present a regression model to estimate the 50th (median) and 99th percentile WSS based on the flow rate and GOA. We examined the 50th percentile instead of the mean because the right-skewed nature of the histogram can result in a large difference between the two, and the knowledge of the WSS threshold which categorizes each half of the cell population is potentially more useful for TEHV maturation protocol development. The 99th percentile data exclude influence from any numerical artifacts along the perimeter, especially near the corners of the leaflets, where the small gap in leaflets does not allow for a sufficient number of boundary layers in the model.

**Figure 8:**
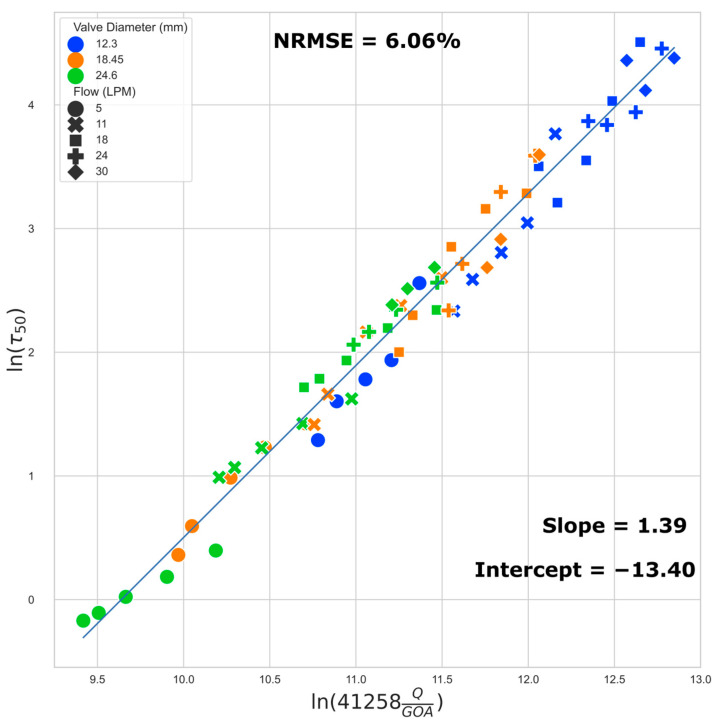
Regression plot: median (50th) percentile WSS. The normalized root mean squared error (NRMSE) value is normalized by range.

**Figure 9:**
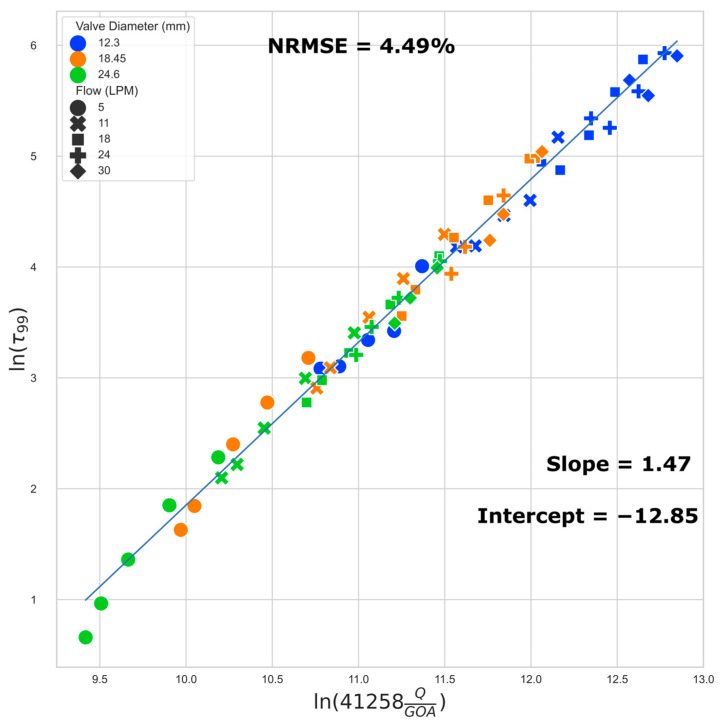
Regression plot: 99th percentile WSS.

The best fit regression lines (Figure 8 and Figure 9) of the transformed WSS (50th and 99th percentile) versus a function of flow rate and GOA clearly show that the regression models are independent of valve size (i.e., the results from all three valve sizes fit to the same regression model). Equations (1) and (2) below describe the regression models for estimating the 50th (median) and the 99th percentile WSS, respectively:

ln(τ_50_) = 1.39 ln(41258 ∗ Q/GOA) − 13.40 (1)

ln(τ_99_) = 1.47 ln(41258 ∗ Q/GOA) − 12.85 (2)

where τ_50_ is the 50th percentile (median) and τ_99_ is the 99th percentile WSS (Pa), Q is the flow rate in m^3^/s, and GOA is the geometric orifice area in m^2^.

**Figure 10:**
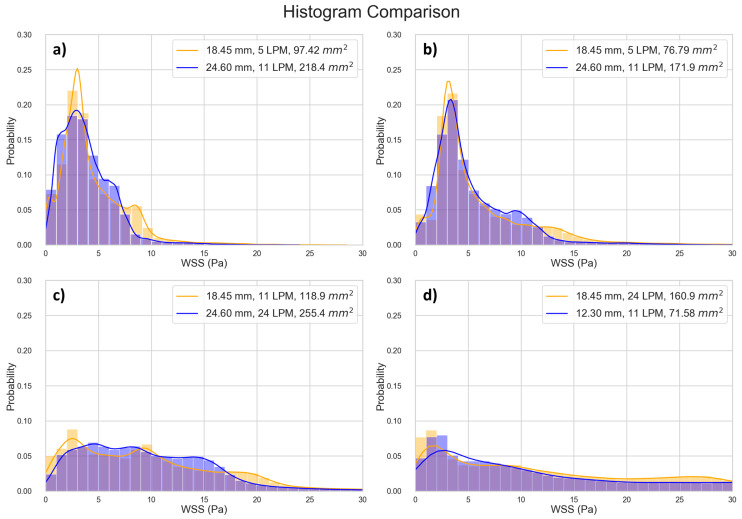
Comparing histograms of selected data points close to each other in Figure 8. (**a**) Point 1 [10.45, 1.227], Point 2 [10.47, 1.231], Percentage Difference [0.179, 0.302]; (**b**) Point 1 [10.69, 1.423], Point 2 [10.71, 1.424], Percentage Difference [0.163, 0.022]; (**c**) Point 1 [11.06, 2.163], Point 2 [11.08, 2.165], Percentage Difference [0.137, 0.063]; (**d**) Point 1 [11.54, 2.338], Point 2 [11.57, 2.333], Percentage Difference [0.261, 0.237].

We found that the data points located close together in Figure 8 also exhibit very similar WSS histograms (Figure 10). 

## 4. Discussion

This research provides useful data quantifying shear stresses in TEHVs in terms of the valve GOA and flow rate. CFD simulations were used to quantify the WSS pattern and magnitude for three sizes of TEHVs. We presented geometrical patterns and histograms of WSS and a regression model to estimate the 50th and 99th percentile WSS based on the GOA and flow rate. 

We identified three regions of WSS variation in the leaflet: high WSS at the tip of the leaflet and the corners of the leaflets, low-to-moderate WSS in the center, and moderate-to-elevated WSS at the base of the leaflets caused by recirculating fluid. These findings agree with previous reports of regional variation in WSS along the length of the valve (Figure 5 in ref [24]). For the smaller valves, at high flow rates, there can be moderate WSS in the center due to detachment of the boundary layer in that region. This knowledge may aid in the design and maturation of TEHVs. For example, one may explore a staged seeding protocol where cells are first seeded in the high WSS areas to begin the maturation process in the bioreactor at a low flow; as the bioreactor flow rate ramps up, additional cells can be seeded in the low WSS area. This could provide the means to apply a more uniform WSS history to all of the cells in the TEHV.

Prior research [20] has reported a WSS of 7.9 Pa on a 23 mm polyurethane valve at a location midway between the leaflet coaptation area and the center of the leaflet, under a flow rate of 22.5 L/min, a fluid viscosity of 3.8 cP, and a density of 1.05 g/cm^3^. In our simulation of the 24.6 mm valve at the fully open position and subjected to a flow rate of 24 L/min, the WSS in the same region was approximately 8 Pa. Another prior investigation [21] reported WSS values of 7.1 Pa in the center for a valve with a 24.8 mm diameter at flow rate of 18 L/min. The fluid used had a dynamic viscosity of 3.5 cP. In our study, the 24.6 mm valve subjected to an 18 L/min flow rate showed WSS of approximately 2.5 Pa at the center of the leaflet. The WSS values we found in our study are similar or slightly lower compared to those in previous reports. Our lower WSS values are likely due to the fact that we considered a fluid with properties resembling water and typical cell culturing media, where the previous studies used fluids with higher viscosity to represent blood. Theoretically, given a specific velocity gradient, WSS scales linearly with the dynamic viscosity. Our results suggest that, with a less viscous fluid, the velocity gradients in our simulation results are similar to those in previous studies, well within one order of magnitude.

In this study, we investigated 66 unique combinations of GOA and flow rates across three different valve sizes. Our results reveal the role of GOA as the primary geometric parameter influencing WSS estimates. This conclusion is supported by both regression equations and the WSS distribution histogram presented in this study. Several key observations substantiate this assertion. Despite the distinct 3D geometries inherent in different-sized valves, the data from valves of varying sizes conform to the same regression line. Furthermore, the WSS regression equations have dependence on only two factors, GOA and the flow rate, with GOA being the only geometric parameter. Finally, the WSS distribution histogram is invariant for scenarios corresponding to closely located points in Figure 8, suggesting a direct connection between the histogram results and their positions on the Figure 8 regression plot. The sensitivity of our reported WSS results exclusively to GOA as a geometric parameter implies that detailed variations in the precise valve shape are not paramount. This insight is important in interpreting and applying our reported data. When using these data to estimate WSS in a bioengineered valve, the exact geometry of the valve need not be identical to that in our simulation for our findings to be relevant and applicable.

The regression models we developed can help tissue engineering researchers quantitatively estimate the WSS a valve is experiencing by measuring the instantaneous flow rate and GOA of the TEHV in a bioreactor, irrespective of the valve size. For example, consider a hypothetical conditioning protocol on a 24 mm valve, where the flow rate is ramped to physiological levels over a 14-day period. Suppose that we measure the peak instantaneous flow rate and the corresponding GOA during the maturation period (Table 3). The 50th and 99th percentile peak stresses can then be estimated via Equations (1) and (2). In this hypothetical conditioning example, we see that on day 5, half of the cells in the TEHV experience peak WSS < 3.39 Pa, and half of the cells experience peak WSS between 3.39 Pa and 13.61 Pa; this scenario corresponds to the location in Figure 8 with x and y values of 10.51 and 1.22, respectively—the closest data point to this location is data point 12 in Table S1, and thus the WSS histogram for the 18.45 mm valve at 62% GOA and 5 LPM (Figure 5) can be used as an estimate. The calculations from the regression equations together with the WSS histogram and WSS geometric patterns provided in this paper enable the tissue engineer to obtain a detailed estimate of WSS levels across the TEHV surface over the maturation time period. 

There are limitations regarding the use of this research’s data. This study considered idealistic aortic valve models; while all aortic vales have similar overall three-leaflet shapes, there may be differences in the geometry between valves, such as the ratio of the length and width of the leaflets. While the valve geometry has the dominate effect on WSS, the surrounding geometry of the bioreactor may have measurable effects; in this study, we considered a standard cylindrical shape of fluid chamber to represent the bioreactor and did not explore varying bioreactor shapes. The data of this study are valid for fluids that resemble the properties of water and standard cell culturing media; if the density and viscosity of the culturing media differ significantly from those used in our simulations, only the qualitative results, such as the WSS geometric patterns and the overall shapes of WSS histograms, may remain useful. Finally, a future experimental validation of WSS in TEHVs under varying bioreactor flow conditions will be useful in corroborating with the results of this study and in solidifying the WSS estimates.

In summary, this study contributes to the field of TEHVs by offering a set of data enabling tissue engineers to estimate WSS in bioreactor valves, a crucial factor for optimizing cell maturation. The dataset includes WSS distribution patterns and histograms, complemented by robust regression models; this information can help shift the current paradigm of largely trial-and-error approaches to more intentional, informed adjustments to bioreactor cell maturation protocols. Our regression models reveal that WSS predominantly depends on the following: (a) GOA rather than the overall valve diameter, highlighting the potential for the application of this study’s results across different valve sizes, and (b) the flow rate, indicating that this commonly measured bioreactor parameter provides critical information for researchers to estimate the WSS stimulus on the valve.

## Figures and Tables

**Table 1:** Summary of geometries selected from FSI simulation results to be used in CFD simulations.

Valve Diameter (mm)	GOA (mm^2^)	% Max GOA
24.60	279.4	100
	255.4	91.41
	218.4	78.17
	171.9	61.52
	129.5	46.35
18.45	160.9	100
	148.7	92.42
	118.9	73.90
	97.42	60.55
	76.79	47.73
12.30	71.58	100
	64.21	89.70
	54.34	75.92
	46.72	65.27
	39.73	55.50

**Table 2:** Combinations of GOA in mm^2^ (selected from FSI simulations), % max GOA (described as a percentage of the maximum GOA measured for each valve size), and flow rates included in the CFD simulations. We did not include the combinations marked with 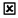 since TEHV maturation conditions typically do not involve the combination of a small GOA and high flow rate.

Valve Diameter (mm)	GOA (mm^2^)	% Max GOA	Flow (L/min)				
			5	11	18	24	30
24.60	279.4	100	✓	✓	✓	✓	✓
	255.4	91.41	✓	✓	✓	✓	✓
	218.4	78.17	✓	✓	✓	✓	✓
	171.9	61.52	✓	✓	✓	✓	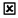
	129.5	46.35	✓	✓	✓	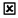	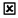
18.45	160.9	100	✓	✓	✓	✓	✓
	148.7	92.42	✓	✓	✓	✓	✓
	118.9	73.90	✓	✓	✓	✓	✓
	97.42	60.55	✓	✓	✓	✓	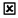
	76.79	47.73	✓	✓	✓	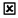	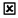
12.30	71.58	100	✓	✓	✓	✓	✓
	64.21	89.70	✓	✓	✓	✓	✓
	54.34	75.92	✓	✓	✓	✓	✓
	46.72	65.27	✓	✓	✓	✓	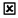
	39.73	55.50	✓	✓	✓	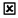	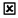

**Table 3:** A hypothetical example to estimate 50th and 99th percentile WSS from the measured flow rate and GOA during a conditioning protocol.

Day	Observation		Estimated Peak WSS (Pa)	
	Peak Flow Rate (L/min)	Maximum GOA (mm^2^)	50th Percentile	99th Percentile
1	5	130	2.13	8.34
2	6.5	150	2.52	9.94
3	8	180	2.61	10.31
4	9.5	195	2.96	11.80
5	11	205	3.39	13.61
6	12	210	3.70	14.92
7	13.5	230	3.84	15.52
8	15	250	3.96	16.03
9	17	260	4.46	18.19
10	18	270	4.58	18.72
11	19.5	275	4.99	20.50
12	21	275	5.53	22.86
13	22.5	278	6.00	24.90
14	24	280	6.50	27.09

## Data Availability

The data supporting the findings of this study are available from the corresponding author upon reasonable request.

## Supplementary Materials

The following supporting information can be downloaded at: https://www.mdpi.com/article/10.3390/jfb15030076/s1, Figure S1: (a) Median (50th percentile), (b) 99th percentile WSS are plotted for successive mesh refinement, quantified in terms of average number of cells per leaflet, for the largest GOA of the 24.6 mm valve. The numbers displayed next to each data point represents the Y axis value; Figure S2: (a) Median (50th percentile), (b) 99th percentile WSS are plotted for successive mesh refinement, quantified in terms of average number of cells per leaflet, for the largest GOA of the 18.45 mm valve. The numbers displayed next to each data point represents the Y axis value; Figure S3: (a) Median (50th percentile), (b) 99th percentile WSS are plotted for successive mesh refinement, quantified in terms of average number of cells per leaflet, for the largest GOA of the 12.3 mm valve. The numbers displayed next to each data point represents the Y axis value; Table S1: Values of data points in Figure 8 and Figure 9. Note that both figures have a common X axis.
