# Supplementary material for: Shear Stress Quantification in Tissue Engineering Bioreactor Heart Valves: A Computational Approach
Source: J Funct Biomater. 2024 Mar 20;15(3):76. doi: 10.3390/jfb15030076 (PMC10971346; doi:10.3390/jfb15030076)
Supplement: Supplementary file 1 [file jfb-15-00076-s001.zip › jfb-2834761-supplementary.pdf]

Supplementary Materials For:

Shear Stress Quantification in Tissue Engineering Bioreactor Heart Valves: A Computational Approach

Grid Independence

The mesh independence study is carried out for the largest GOA corresponding to each valve for a flow of 30 LPM. The figures S1, S2 and S3 summarize the results of consecutive mesh refinement on the 50th (median) and 99th percentile WSS for valves 24.6, 18.45 and 12.3 mm respectively. The meshing parameters from the circled data points are utilized for meshing other GOAs of the corresponding valve size.

We evaluated six meshes ranging from 5x10^3 to 5.7x10^4 elements per leaflet (total number of elements ranging from 1.4 to 7.5 million) for the 24.6 mm diameter valve. The results are considered converged when there is less than 5% and 10% change in the 50th and 99th percentile WSS respectively. The meshing parameters that met the convergence criteria resulted in a mesh with 5.3 million total elements (fig. S1). Three meshes were evaluated for the 18.45 mm valve and less than 3% change in the quantities of interest were observed (fig. S2). Therefore, the parameters from the third mesh resulting in a total of 5.79 million total elements were selected for all 18.45 mm valve simulations. Seven meshes ranging from 1.1x10^4 to 3.4x10^4 elements per leaflet (total elements ranged from 3.6 to 12 million) are evaluated for the 12.3 mm diameter valve, where each successive mesh resulted in an average of a 20% increase in the number of cells per leaflet (fig S3). Comparing the 6th and 7th data points in fig. S3 with the 5th (circled), the change observed in 50th percentile WSS is -4% and 8.7%, while the change in 99th percentile WSS is -6.5% and 2.4%. These do not meet the criteria for median WSS of less than 5% but the successive mesh resulted in doubling of the total number of elements as well as doubling of the simulation computing time from 22 to 44 hours. Thus, practical considerations limited us to meshing parameters containing 2.3x10^4 elements per leaflet and a total of 5.9 million elements.

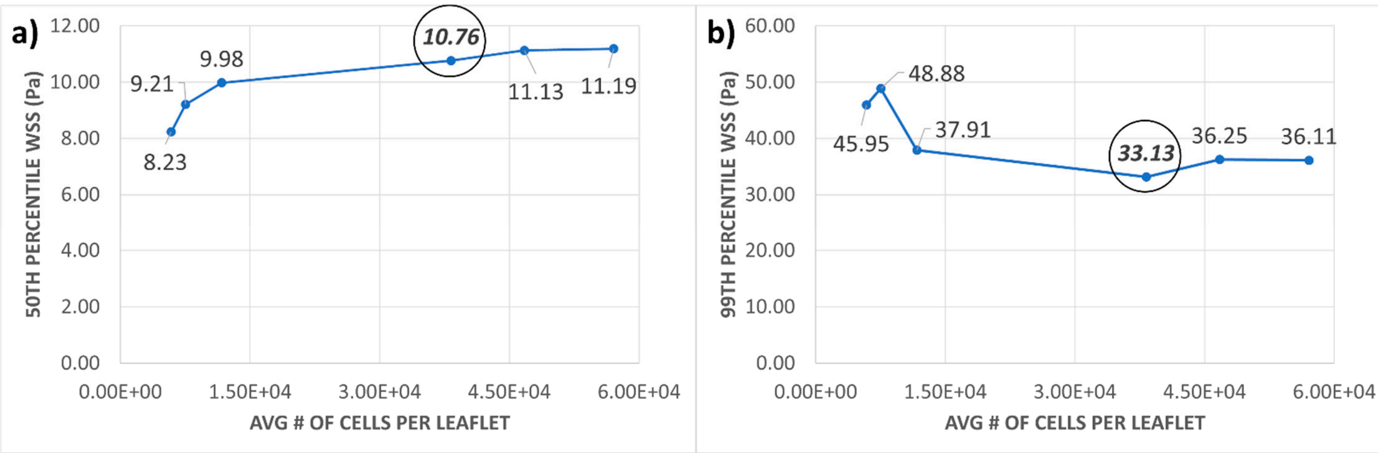

Figure S1: a) Median (50th percentile), b) 99th percentile WSS are plotted for successive mesh refinement, quantified in terms of average number of cells per leaflet, for the largest GOA of the 24.6 mm valve. The numbers displayed next to each data point represents the Y axis value.

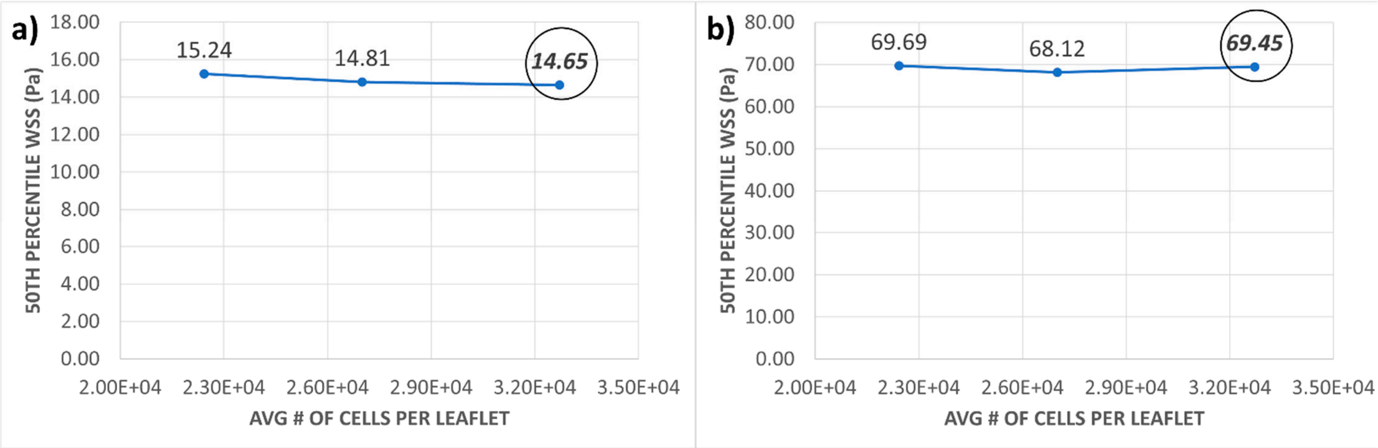

Figure S2: a) Median (50th percentile), b) 99th percentile WSS are plotted for successive mesh refinement, quantified in terms of average number of cells per leaflet, for the largest GOA of the 18.45 mm valve. The numbers displayed next to each data point represents the Y axis value.

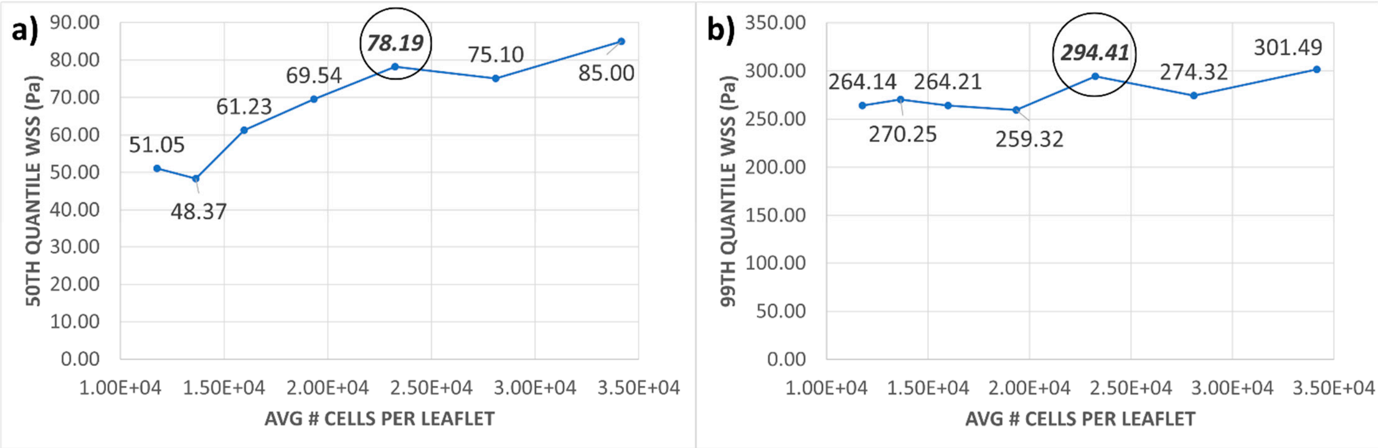

Figure S3: a) Median (50th percentile), b) 99th percentile WSS are plotted for successive mesh refinement, quantified in terms of average number of cells per leaflet, for the largest GOA of the 12.3 mm valve. The numbers displayed next to each data point represents the Y axis value.

Index	Flow (LPM)	GOA (mm^2)	Valve Diameter (mm)	Data X Axis	Data Y Axis (median)	Data Y Axis (99th percentile)
1	5	279.4	24.60	9.418	-0.170	0.659
2	5	255.4	24.60	9.507	-0.107	0.965
3	5	218.4	24.60	9.664	0.022	1.361
4	5	171.9	24.60	9.903	0.184	1.851
5	5	160.9	18.45	9.969	0.362	1.629
6	5	148.7	18.45	10.05	0.594	1.845
7	5	129.5	24.60	10.19	0.397	2.283
8	11	279.4	24.60	10.21	0.989	2.098
9	5	118.9	18.45	10.27	0.986	2.401
10	11	255.4	24.60	10.30	1.069	2.219
11	11	218.4	24.60	10.45	1.227	2.547
12	5	97.42	18.45	10.47	1.231	2.778

13	11	171.9	24.60	10.69	1.423	2.996
14	18	279.4	24.60	10.70	1.717	2.778
15	5	76.79	18.45	10.71	1.424	3.180
16	11	160.9	18.45	10.76	1.415	2.908
17	5	71.58	12.30	10.78	1.290	3.084
18	18	255.4	24.60	10.79	1.786	2.979
19	11	148.7	18.45	10.84	1.661	3.091
20	5	64.21	12.30	10.89	1.603	3.104
21	18	218.4	24.60	10.95	1.933	3.224
22	11	129.5	24.60	10.98	1.623	3.406
23	24	279.4	24.60	10.99	2.061	3.207
24	5	54.34	12.30	11.06	1.781	3.342
25	11	118.9	18.45	11.06	2.163	3.546
26	24	255.4	24.60	11.08	2.165	3.460
27	18	171.9	24.60	11.18	2.196	3.662
28	5	46.72	12.30	11.21	1.936	3.423
29	30	279.4	24.60	11.21	2.383	3.493
30	24	218.4	24.60	11.23	2.344	3.722
31	18	160.9	18.45	11.25	2.001	3.558
32	11	97.42	18.45	11.26	2.377	3.895
33	30	255.4	24.60	11.30	2.514	3.721
34	18	148.7	18.45	11.33	2.299	3.795
35	5	39.73	12.30	11.37	2.559	4.008
36	30	218.4	24.60	11.46	2.686	3.993
37	18	129.5	24.60	11.47	2.342	4.098
38	24	171.9	24.60	11.47	2.563	4.052
39	11	76.79	18.45	11.50	2.602	4.294
40	24	160.9	18.45	11.54	2.338	3.940
41	18	118.9	18.45	11.55	2.853	4.265
42	11	71.58	12.30	11.57	2.333	4.182
43	24	148.7	18.45	11.62	2.715	4.182
44	11	64.21	12.30	11.68	2.589	4.190
45	18	97.42	18.45	11.75	3.160	4.602
46	30	160.9	18.45	11.76	2.685	4.241
47	30	148.7	18.45	11.84	2.913	4.476
48	24	118.9	18.45	11.84	3.296	4.645
49	11	54.34	12.30	11.84	2.806	4.465
50	18	76.79	18.45	11.99	3.285	4.978
51	11	46.72	12.30	11.99	3.046	4.601
52	24	97.42	18.45	12.04	3.589	5.002
53	18	71.58	12.30	12.06	3.503	4.949
54	30	118.9	18.45	12.06	3.597	5.040
55	11	39.73	12.30	12.16	3.766	5.171
56	18	64.21	12.30	12.17	3.209	4.874
57	18	54.34	12.30	12.34	3.551	5.189
58	24	71.58	12.30	12.35	3.869	5.341
59	24	64.21	12.30	12.46	3.837	5.256
60	18	46.72	12.30	12.49	4.031	5.578
61	30	71.58	12.30	12.57	4.359	5.685

62	24	54.34	12.30	12.62	3.941	5.585
63	18	39.73	12.30	12.65	4.507	5.872
64	30	64.21	12.30	12.68	4.117	5.546
65	24	46.72	12.30	12.77	4.456	5.930
66	30	54.34	12.30	12.85	4.379	5.904

Table S1: Values of data points in figures 8 & 9. Note that both figures have a common X axis
